# The Severity of Neurological Dysfunction in Preschool Children, Secondary to Damage Generated During the Perinatal Period, is Associated With a Pro-Inflammatory Pattern of Serum Molecules

**DOI:** 10.3389/fimmu.2020.595309

**Published:** 2021-01-28

**Authors:** Miriam Madrid, Malinalli Brianza-Padilla, Juan C. Echeverría, Rolando Rivera-González, Rafael Bojalil

**Affiliations:** ^1^ Doctorado en Ciencias Biológicas y de la Salud, Universidad Autónoma Metropolitana, Mexico City, Mexico; ^2^ Department of Immunology, Instituto Nacional de Cardiología Ignacio Chávez, Mexico City, Mexico; ^3^ Department of Electric Engineering, Universidad Autónoma Metropolitana-Iztapalapa, Mexico City, Mexico; ^4^ Neurodevelopment Monitoring Laboratory, Instituto Nacional de Pediatría, Mexico City, Mexico

**Keywords:** perinatal risks, inflammation, sequelae, neurological signs, neurodevelopment

## Abstract

Disorders in the child’s neurological development caused by perinatal risks can lead to long-term altered neurological signs that begin at an early age and involve persistent functional disorders. Recent data suggest that tissue dysfunction, not just acute damage, may initiate or perpetuate an inflammatory response. The aim of this study was to find out if any neurological dysfunction in preschool children secondary to damage generated during the perinatal period is associated with the magnitude of perinatal risks and long-term modifications in the serum concentrations of inflammatory molecules. The participants, aged 1–4 years, were on neurodevelopmental follow-up and rehabilitation therapy from the first three months of life and had no acute disease data. We classified the children into three groups according to the importance of their perinatal risks: low, medium, and high. The results show that 1) the magnitude of perinatal risks correlated with the severity of neurological dysfunction; 2) the greatest changes in the concentrations of the molecules of the inflammatory process were associated with the most altered neurological signs. This suggests that persistent nervous system dysfunction keeps inflammatory responses active even in the absence of an acute process of infection or damage.

## Introduction

A history of perinatal risks, from 22 weeks of gestation to 28 days after birth, increases the likelihood of persistent altered neurological signs initiated at an early age (1–3). For example, perinatal asphyxia in full-term infants is the leading cause of brain damage and neurological sequelae, responsible for motor, sensory, and cognitive disorders, with high individual, family, and social costs (4, 5). Prematurity (6), infections (7), or endocrine disorders (8) can also have a negative impact on neurogenesis, neuronal migration, axon and dendrite formation, synaptogenesis, myelinization, and regulation of specific neurotransmitters (7). Thus, knowledge of the impact of perinatal risks on neurological development allows us to focus on preventive measures and interventions needed to minimize sequelae: the earlier the intervention, the better the outcome (9, 10).

The impact of the perinatal risks explains the potential features of the disruptions in the child’s neurodevelopment and the sequelae it entails (1). Normal function may be achieved if homeostatic adjustments to harmful conditions induced by perinatal risks meet the needs of the organs. If the body’s resilience or interventional therapy compensate for a functional impairment, the subject will remain with a systemic functioning that, although forced, may even resemble normal functionality (11–13). However, perinatally acquired damage can cause a chronic neurophysiological disorder in which new parameters of functioning take place (14–17). These sequelae may be evident (e.g. motor or severe cognitive abnormalities) or not (e.g. inadequate responses to stimuli) (18), either way implying functional disarrays. Recent data suggest that tissue dysfunction, and not only acute damage, can act as an inflammatory trigger. Therefore, even when the primary harmful event has long since disappeared, a perpetual cycle of inflammation-dysfunction-inflammation may occur in the form of low-grade chronic inflammation or chronic para-inflammation (19, 20), with systemic repercussions (21–23). Furthermore, any abnormal condition that occurs in the structures of the central nervous system can alter not only its functioning, but also that of other structures that also participate in the response to different intrinsic or extrinsic challenges (24). Namely, the functioning of the vagus nerve, which has a regulatory activity on the inflammatory process (25–29). We hypothesized that the persistence of neurological signs, regulatory disorders, or motor syndromes could be reflected in different patterns of modifications of biomarkers of the inflammatory process.

Thus, the aim of this study was to find out if in preschool children both the magnitude of their perinatal risks and the presence and severity of the derived neurological dysfunctions are associated with long-term modifications in the serum concentrations of the molecules of the inflammatory process.

## Materials and Methods

This was an observational and cross-sectional study. It involved the measurement of neurological signs in 45 children aged 1 to 4 years. Specialists from the Neurodevelopment Laboratory at the Instituto Nacional de Pediatría (National Institute of Pediatrics) of Mexico City carried out the neurological examination within the follow-up of neurological development, which included the description and measurement of tone quality, pathological reflexes, pyramidal signs, asymmetries, and dysautonomia. We carried out the evaluation by items that we recorded in a database. We followed all children from the first 3 months of life, and we performed the evaluations according to the follow-up protocol, every 3 months. All children came to our facility every 15 days for group interventions offered by highly qualified staff; if during follow-up the children’s development was delayed or worsened, then we performed individual interventions every week until they were back on track. Children in our school-going cohorts maintain adequate functional standards to attend without special attention. All the children’s parents or legal guardians signed an informed consent letter that was explained in detail. This letter described the number of blood samples needed, the measurements to be taken, and the use of all data collected. The letter also explained that participation was voluntary, as well as the possibility of withdrawing from the study at any time without consequences for their care. The research and ethics committees of the Instituto Nacional de Cardiología (National Heart Institute) approved the research protocol (# 17-1032).

### Inclusion Criteria

All the children belonging to our cohorts who met the age parameter (1–4 years old) and who had their first neurodevelopmental evaluation and follow-up according to the Neurodevelopment Laboratory protocol. They had to be active Laboratory attendees as part of a cohort. Our cohorts included children with low, medium, and high perinatal risk of neurodevelopmental disorders. In the high-risk group, we included children who had had moderate perinatal asphyxia, with a stay in the intensive care unit, and follow-up by specialists until discharge. For moderate risk, we included children with perinatal factors such as prolonged expulsion periods or moderate prematurity; we also included children with a history of threatened preterm birth or children with congenital cardiac disorders who could be followed from home. None of them required extraordinary interventions to keep them alive, even if they had short stays in the intensive care unit. For the low risk children, it was not necessary for them to be in an intensive care unit, they had no congenital disorders and they were discharged from the hospital with their mother after birth (9).

### Exclusion Criteria

All children with any genetic syndrome, with cerebral palsy or seizure syndrome, with any chronic disease that can cause prolonged inflammatory processes (such as cancer, asthma, and allergies). Major surgical procedures, fractures, or head trauma occurring within 6 months prior to blood sampling.

### Elimination Criteria

Parents’ decision to exclude their child from the protocol, accidents with associated inflammation, surgical procedures after the first evaluation.

### Blood Sample Collection

We collected 5 to 10 ml of peripheral blood; then we separated the serum, made aliquots and froze them at -70C until use. We collected all samples between February and March 2018. We asked parents to avoid giving their children any neurostimulator such as chocolate or coffee during the previous 24 h. We also asked them to give them a light breakfast consisting of a glass of milk and half a portion of fruit eaten 4h before the sample was taken. If we detected an acute disease, we postponed the appointment for two weeks. We were not able to obtain blood samples from all the children; thus, we report more neurological signs than inflammatory molecules.

### Detection of the Molecules of the Inflammatory Process

Most of the protein molecules were measured by the Affymetrix by eBioscience *Human Inflammation Panel* (20 plex) multiplex system on a Luminex MAGPIX System (Luminex Corporation). The analytes and their detection ranges were: GM-CSF (18–73,300 pg/ml), IFN-gamma (11–11,075 pg/ml), TNF-alpha (6.98–28,600 pg/ml), IL-10 (2.49–10,200 pg/ml), IL-12p70 (6.67–6,825 pg/ml), IL-13 (2.59–10,600 pg/ml), IL-17A (2.27–9,300 pg/ml), IL-1beta (2.09–8,550 pg/ml), IL-4 (11–45,200 pg/ml), IL-6 (11–10,850 pg/ml), sICAM-1 (212–870,200 pg/ml), E- Selectin (250–255,500 pg/ml), P-Selectin (1,233–5,051,600 pg/ml), IFN-alpha (0.49–2,000 pg/ml), IL-1alpha (0.55–2,250 pg/ml), IL-8 (2.47–10,100 pg/ml), IP-10 (1.25–5,100 pg/ml), MCP-1 (1.15–4,700 pg/ml), MIP-1alpha (1.86–1,900 pg/ml), MIP-1beta (4.22–17,300 pg/ml).

We used ELISA kits to determine Resolvin D1 (RvD1, Cayman Chemical, assay range of 3.3–2,000 pg/ml and sensitivity approximately 15 pg/ml), Lipoxin A4 (LXA4, Cloud-Clone Corp. CEB452Ge, detection range 493.8–40,000 pg/ml, minimum detectable is typically less than 166.1 pg/ml), Leukotriene B4 (LTB4, R&D Systems KGE006B, sensitivity 10.9 and assay range 10.3–2,500 pg/ml), Annexin A1 (Cayman Chemical, 501550, range of 0.20–20 ng/ml, sensitivity 0.2 ng/ml and lower limit of detection 0.18 ng/ml).

Of all the measured molecules, only those with statistical significance were included in the results tables.

### Statistics

We transformed the statistical data on the concentrations of inflammatory molecules into standardized values with a mean of 0 and a standard deviation of 1 to express them as Z-scores. Since we found no differences by age and sex, no adjustments were necessary for these variables. We compared perinatal risk and neurological signs variables with standardized values of the inflammatory molecules. We compared the means of more than two groups by the analysis of variance (ANOVA) and a *post hoc* Tukey-Kramer test. If the data did not meet the parameters of homogeneity of variance and normal distribution, non-parametric statistics were performed with the Wilcoxon test. Due to the sample size and the dispersion of the groups, we considered that the differences were significant when the probability of error was p <0.05 and marginally significant when it was between 0.05 and <0.1. If the latter was the case, we additionally made univariate comparisons to assess significance.

## Results

### Study Population

We studied a total of 45 children (20 female and 25 male) from 1 to 4 years old. For their follow-up we divided the children into three groups according to their perinatal risks: low, medium, and high, which were classified according to our charted instrument (9) placing 10, 11, and 24 children in the low, medium and high-risk groups, respectively. All children included in the high perinatal risk group had a history of perinatal asphyxia, all of them treated with 72 h hypothermia.

### Alterations of Neurologic Signs in Preschool Children Varied According to the Magnitude of their Perinatal Risks

#### Tone Alterations

About 60% of all the children had tone disorders. However, we found large differences between perinatal risk groups in the type and degree of tone alterations. Hypotonia, for example, was the most frequent disorder in all three groups but its proportion decreased as perinatal risk increased. The opposite occurred with hypertonia, which proportion increased in parallel with the magnitude of perinatal risk. Only in the high-risk group we found children with fluctuations in tone, which we consider severe due to its association with alterations in various neurological areas. Regarding the degree of tone alterations, none of the low-risk children presented severe disorders, while almost 10% of the medium- and high-risk children did (**Table 1**).

**Table 1:** Neurological Signs in Preschool Children by Group of Perinatal Risks.

Neurological Signs	Perinatal Risks Groups		
	Low n (%)	Medium n (%)	High n (%)
Alteration of tone • No • Hypotonia • Hypertonia • Fluctuation Degree Mild Moderate Severe	4 (40%) 5 (50%) 1 (10%) 0 5 (50%) 1 (10%) 0	5 (45%) 4 (37%) 2 (18%) 0 3 (28%) 2 (18%) 1 (9%)	10 (42%) 8 (33%) 5 (21%) 1 (4%) 10 (42%) 2 (8%) 2 (8%)
Pathological Reflex Activity No Mild Moderate	10 (100%) 0 0	9 (82%) 2 (18%) 0	18 (75%) 4 (17%) 2 (8%)
Pyramidal Reflex No Discrete Evident	10 (100%) 0 0	9 (82%) 2 (18%) 0	21 (87%) 2 (9%) 1 (4%)
Dysautonomia No Emotional Fluctuating tone Sensorial Degree Mild Moderate Severe	4 (40%) 6 (60%) 0 0 1 (0%) 4 (40%) 1 (10%)	7 (64%) 4 (36%) 0 0 0 4 (36%) 0	7 (29%) 15 (63%) 1 (4%) 1 (4%) 6 (24%) 8 (36%) 3 (11%)
Asymmetries No Yes	5 (50%) 5 (50%)	7 (64%) 4 (36%)	6 (25%) 18 (75%)

#### Pathological Reflex Activity

Children with low perinatal risk showed no pathological reflex activity; the degree of alteration increased in the other two groups. Within the medium perinatal risk, about one fifth of this group had this sign to a mild degree; and children with this sign in high perinatal risk (about one-quarter) had a 2:1 distribution to mild and moderate degrees, respectively (**Table 1**).

#### Pyramidal Signs

No child with low perinatal risk presented pyramidal signs. Those who had them in the medium-risk group presented discrete pyramidal signs, and in the group of children with high-perinatal risk we found a 2:1 distribution of discrete and evident pyramidal signs, respectively (**Table 1**).

#### Dysautonomia

In the children with low and medium risks, the dysautonomia was in all cases of an emotional type. In high-risk children, one had fluctuation of the tone and another of a sensory type (**Table 1**).

#### Asymmetries

The group with the most frequent cases of asymmetries was the high-risk group. Notably, we found the least frequent cases of asymmetries in the medium risk group (**Table 1**).

### The Molecules of the Inflammatory Process Varied According to the Magnitude of the Perinatal Risks

**Figure 1:**
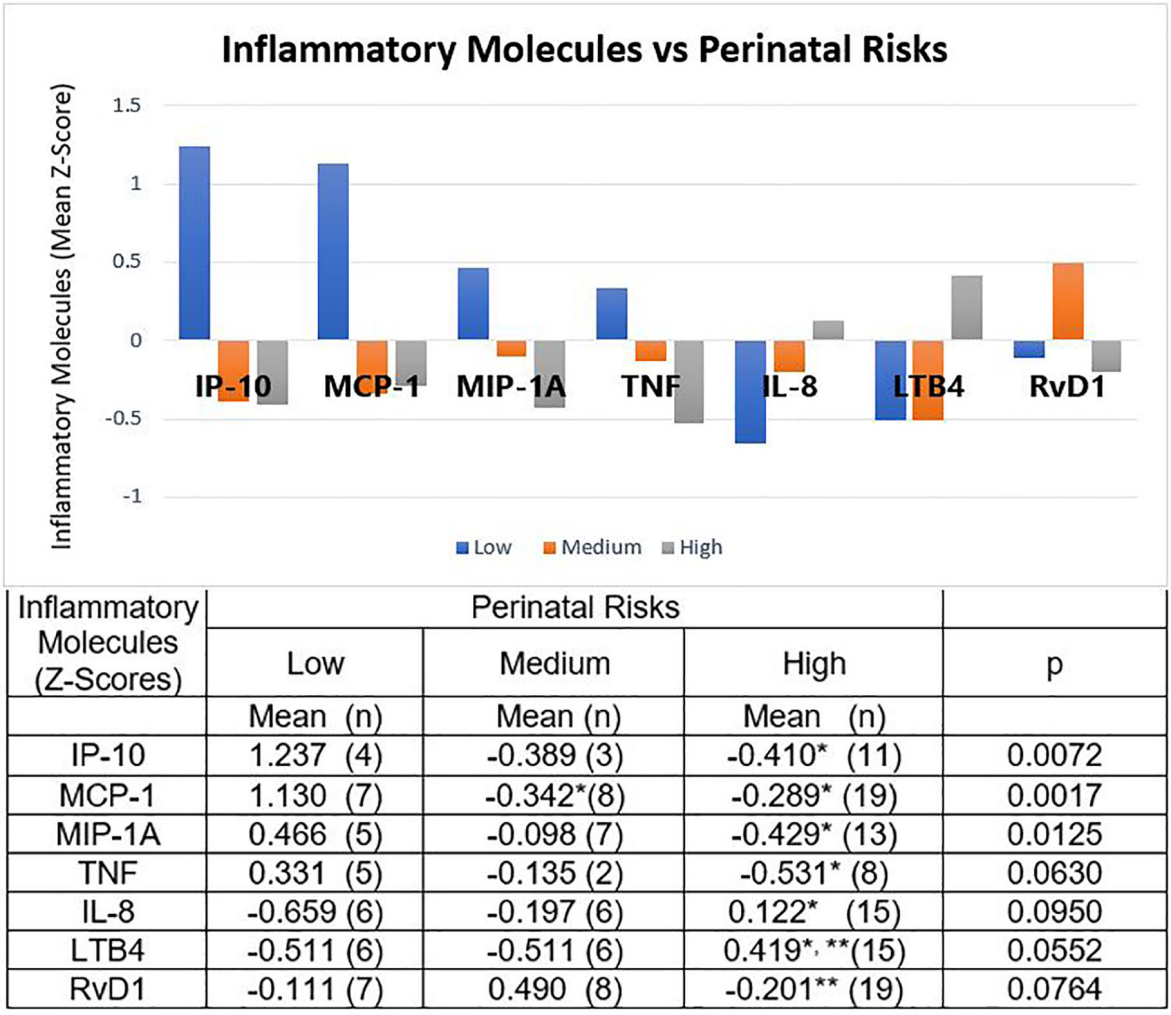
Molecules of the Inflammatory Process and Perinatal Risks. Children at low perinatal risk had positive Z-scores for mean concentrations of IP-10, MCP-1, MIP-1A, and TNF; and negative Z-scores for IL-8, LTB4, and RvD1. Medium perinatal risks group had negative Z-scores for all these molecules, except RvD1. The Z-scores of the children with high perinatal risk had the opposite sign to that of the low risk group, except for RvD1. The table expresses the complete numerical data and the significances across the groups and the paired comparisons. *p < 0.01 vs low-risk group; **p < 0.05 vs medium-risk group.

The Z-scores of the mean concentrations of IP-10, MCP-1, MIP-1A, and TNF were positive for the low risk group and negative for the other two groups, reaching a significance across the perinatal-risk groups for the first three, and marginal significance for TNF. In paired comparisons with the low-risk group, the medium-risk group had only significantly lower means of MCP-1; the high-risk group had significantly lower means of IP-10, MCP-1, MIP-1A, and TNF (**Figure 1**).

In contrast, Z-scores were negative for chemokine IL-8 and proinflammatory lipid mediator LTB4 for low and medium risk groups, and positive for the high-risk group, being marginally significant across the groups. Paired comparisons showed statistical significance for IL-8 between high- and low-risk, and for LTBA between high-risk and the other two groups.

The behavior of the resolution lipid mediator RvD1 was particular. For this molecule we found a positive Z-score for the medium risk group and negative Z-scores for the low- and high-risk groups, with marginal significance across the groups. Paired comparisons showed significance when comparing the high-risk group with the medium-risk group (**Figure 1**).

### The Molecules of the Inflammatory Process Varied According to the Type and Degree of Alterations of the Tone

We found a trend towards higher mean concentrations of GM-CSF, IL-8, IFN-α, IL-6, IL-17A, IL-18, and IL-10, when there were alterations of the tone compared to their absence. The trend for IFN-γ and RvD1 was the opposite; however, only IL-18 reached significance (p < 0.0325). (data not shown)

**Figure 2:**
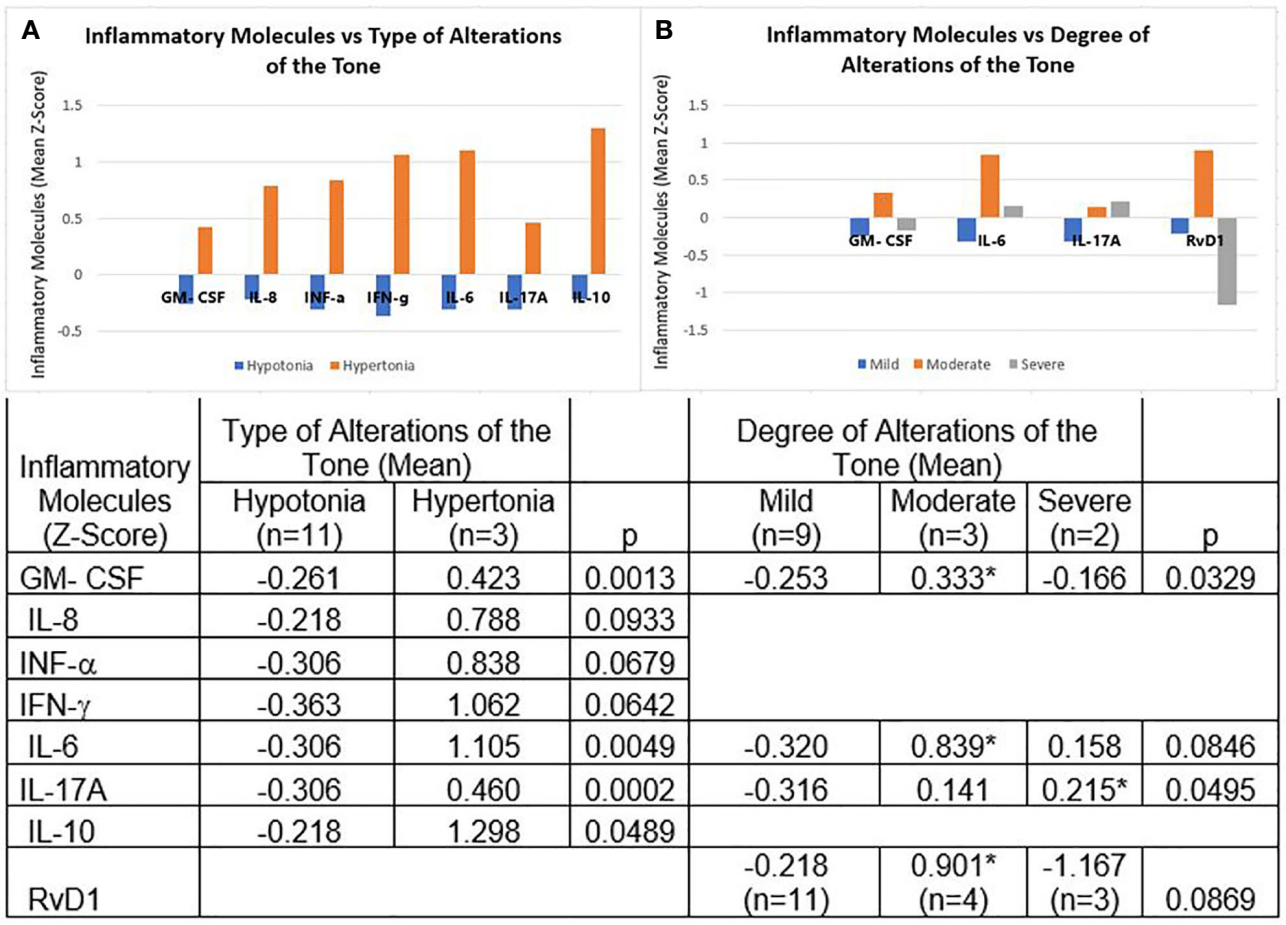
Molecules of the Inflammatory Process and Tone Alterations. **(A)** Hypotonia vs hypertonia. The Z-scores of the means of GM-CSF, IL-6, IL-17A, IL-10, IL-8, IFN-α, and IFN-γ were all positive in hypertonia and negative in hypotonia. **(B)** Degrees of tone alterations. In the mild degree group, Z-scores were all negative for GM-CSF, IL-6, IL-17A, and RvD1; in the moderate degree they were all positive. In the severe degree group, IL-6 and IL-17A were positive and GM-CSF and RvD1 were negative. The table expresses the complete numerical data and the significances across the groups and the paired comparisons. *p < 0.05 vs mild degree group.

In the case of hypotonia vs hypertonia, the Z-scores of the means of GM-CSF, IL-6, IL-17A, IL-10, IL-8, IFN-α, and IFN-γ were all positive in the latter, and negative in the hypotonia, with the first four reaching statistical significance (**Figure 2**, panel **A**).

As for the degrees of tone alterations, the Z-scores were all negative for GM-CSF, IL-6, IL-17A, and RvD1 in the mild degree group, and all positive in the moderate degree group. In the severe degree group IL-6 and IL-17A were positive and GM-CSF and RvD1 negative. Across the groups we found significant differences for GM-CSF and IL-17A, and marginal significance for IL-6 and RVD1. In paired comparisons, GM-CSF, IL-6, and RvD1 concentrations were significantly higher in children with moderate impairment in tone than in those with mild impairment; IL-17A was significantly elevated in severe impairment in tone compared to mild impairment (**Figure 2**, panel **B**).

### The Molecules of the Inflammatory Process Varied With the Presence of Asymmetries

We found a marginal significance towards a higher concentration of molecules related to the inflammatory response associated with innate immunity in children with asymmetries. These included adhesion molecules (sICAM), chemokines (MIP-1A, IL-8), growth factors (GM-CSF), and cytokines (IL-1α, IL-6); we also found IL-13 (observed in later stages - adaptive immunity), and RvD1, the pro-resolving molecule, with marginal significance towards higher concentrations (data not shown).

### The Molecules of the Inflammatory Process Varied According to the Presence and Degree of Pathological Reflex Activity

**Figure 3:**
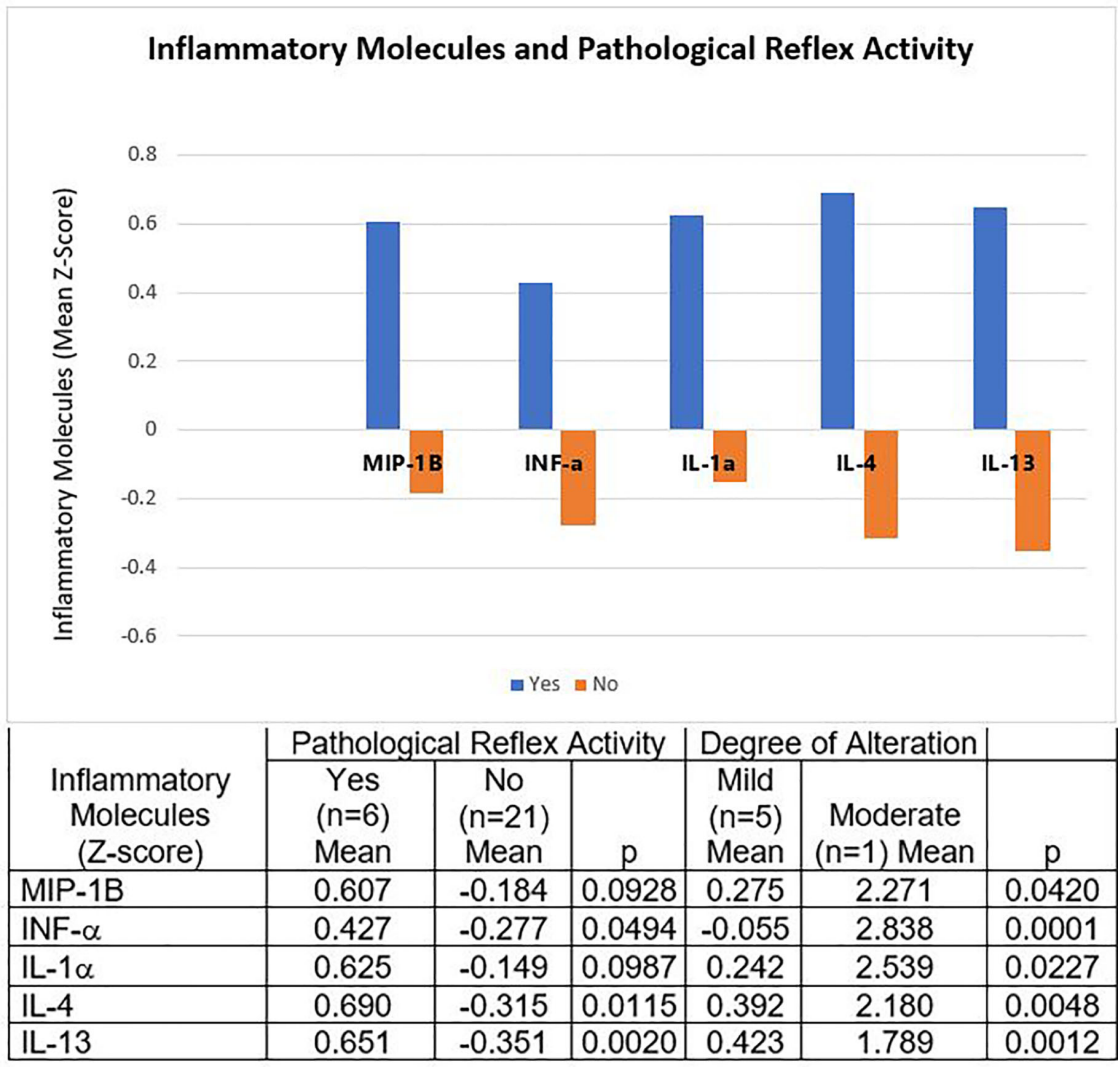
Molecules of the Inflammatory Process and Pathological Reflex Activity. Children with pathological reflex activity had positive Z-scores for the means of IFN-α, IL-1α, MIP-1B, IL-4, and IL-13. The Z-scores of all these mediators were negative in the children without pathological reflex activity. The only child with moderate degree pathological reflex activity had much higher concentrations of all these molecules than the mild degree. The table expresses the complete numerical data and the significances across the groups and the paired comparisons.

In children with pathological reflex activity we found positive Z-scores for the means of IFN-α, IL-1α, MIP-1B, IL-4, and IL-13, in contrast to negative Z-scores for all these mediators in children without pathological reflex activity. As for the molecules expressed mainly during the early stages of inflammation and innate immunity, we found significantly elevated concentrations of IFN-α and those of IL-1α and MIP-1B with marginal significance. We also found significantly elevated the Th2-type cytokines IL-4 and IL-13, all of them compared to those without pathological reflex activity (**Figure 3**).

The only child with a moderate degree of pathological reflex activity had much higher concentrations of the aforementioned molecules than the mild-degree ones (**Figure 3**).

## Discussion

In the perinatal stage, the brain is critically vulnerable to a variety of biological and environmental factors that may interfere with the normal development of its structures and functionality. This implies that adequate and detailed perinatal surveillance and interventions will influence the outcome related to neurodevelopmental disorders (30–34). In this study, the various alterations of neurological signs in preschool children varied according to the magnitude of their perinatal risks. The greatest number and severity of neurological signs occurred in children with a high-risk perinatal history of asphyxia. In contrast, as expected, children with low-perinatal risks achieved better neural maturation. For example, in the high-risk group some children presented sensory dysautonomia or fluctuating tone (8%), pathological reflex activity (8%), or evident pyramidal signs (4%), contrasting to none in the other two groups. This possibly reflects incorrect functioning of structures such as the brain stem, limbic system and hypothalamus (24), or damage to the pyramidal pathway (35). Similarly, high-risk (8%) and medium-risk (9%) children had severe alterations in tone. This could reflect an inadequate functioning of the upper motor neuron and its interaction with the cerebellum. The latter because of its influence on the medial descending system involved in the regulation of muscle tone, posture, spinal reflexes, balance, and the execution of fine movements (36–38). Asymmetry in childhood is a clinical picture involving posture, function, and movements. Although the etiologies of asymmetries are usually of biological origin, many of them are related to environmental and upbringing factors (39). Asymmetries occurred in large numbers in all the perinatal risk groups: half of the low-risk group, a third of the medium-risk group, and three quarters of the high-risk group. One possible explanation for this finding could be the very low formal education of the parents of the children in our cohorts. The fact that the medium-risk children received the same therapeutic interventions as high-risk children may explain their better performance.

Consistent with our hypothesis, we found that the most altered neurological signs were associated with the greatest changes in the serum concentrations of the molecules of the inflammatory process. These findings are also consistent with the postulate that inflammation results not only from the presence of microorganisms or acute tissue damage, but also from its modified functions (23). Furthermore, an inflammatory state could reinforce the dysfunction, perpetuating the process. Thus, whatever the cause of the altered function, if not corrected, it can lead to chronic inflammation and eventually to systemic repercussions (40–42). Perinatal hypoxia and other perinatal risks leading to sequelae could be a good example of this phenomenon.

It has been documented that cytokine producing cells express receptors for neurotransmitters, which makes the inflammatory response subject to nervous system modulation (18). In parallel, local or peripheral concentrations of cytokines can influence the responses of the central nervous system (26). Thus, it is conceivable that a chronic dysfunction of the central nervous system may modify the parameters of the inflammatory response and, in turn, that a chronic inflammatory response, even if subtle, may influence the functioning of the central nervous system. In this work, when we assessed the molecules of the inflammatory process according to the magnitude of the perinatal risk, we found that preschool children with high perinatal risk had higher concentrations of chemokine IL8 (CXCL8) than children with low risk. These same children had lower concentrations of the chemokines IP-10, MCP-1, and MIP-1A, and the pro-inflammatory cytokine TNF than the children at low risk. We would like to draw special attention to lipid mediators. In high-risk children we found high concentrations of LTB4, a pro-inflammatory molecule that originates from arachidonic acid. This lipid mediator also participates in the induction of hyperalgesia (43) with the participation of neutrophils (44), which are attracted and activated by IL-8 and LTB4. In sharp contrast, we found that the pro-resolving lipid mediator RvD1 was elevated in the medium risk group reaching significance when compared to the high-risk group, as if the resolution and repair mechanisms were active in mild conditions but exhausted when the damage is deep (40). These data suggest long-lasting molecular effects of perinatal risks, despite successful neurodevelopmental interventions.

We also assessed the molecules of the inflammatory process by the presence and severity of different neurological signs (tone disorders, asymmetries, and pathological reflex activity), regardless of the magnitude of perinatal risks. We found that a dysfunctional nervous system was associated with an active production of inflammatory mediators, manifested in a differential presence of molecules from both the proinflammatory and the anti-inflammatory/pro-resolving phases. Notably, we found some differences in the profiles of these molecules when comparing the presence or absence of a given neurological sign; nonetheless, the most acute differences were found when evaluating the actual severity of the sign.

Regarding tone disorders, we found significantly higher levels of proinflammatory mediators (GM-CSF, IL-6, and IL-17A), and the anti-inflammatory IL10, in children with hypertonia than in those with hypotonia. Children with moderate severity of tone disorders had higher concentrations of IL-6 and RvD1 than children with severe disorders, the most striking finding being that the pro-resolving molecule had very low concentrations in the latter, which resembles the results of children with higher perinatal risks. During inflammation, the signaling pathway activated by prostaglandins (PGE2 and PGD2) initiates the transcription of the enzyme 15-Lipoxygenase, necessary for the generation of lipoxins, as well as resolvins and protectins, as a signal to initiate the resolution of inflammation (45). However, as an effect of chronic low-grade inflammation, it is likely that the stimuli for resolvin production are overwhelming and therefore their production declines, or that the excessive consumption of these lipid mediators exceeds their production.

A disorder in the organization of reflex activity is evidence of an inappropriate maturation of brain structures and responses to different stimuli (7). Our findings show that this disorder may also be associated with high concentrations of various mediators of the immune/inflammatory response, some observed in its early phases (INF-α, IL-1α, and MIP-1B), and others observed later (IL-4, IL-13). The concentrations of these molecules were significantly higher in children with a moderate alteration in their reflexes compared to a mild alteration. The autonomic nervous system links the immune activity and the nervous system. The efference of the sympathetic and parasympathetic nerves innervate lymphoid tissues such as the thymus, bone marrow, spleen, lymph nodes, and the gut associated lymphoid tissue. This may explain the mechanisms by which altered pathological reflex activity does not mitigate the formation of inflammatory molecules (46).

Asymmetries occur in a large part of the population because, although they may be of organic origin, they are also determined, as mentioned, by upbringing and other external factors. Thus, one explanation for the marginally significant inflammatory activity found here in children with asymmetries could be that this sign is not necessarily fully explained by damage of perinatal origin.

Further studies are granted to reinforce our findings, consistent with the postulation that tissue dysfunction generates a subclinical inflammatory response that often goes unnoticed. This would be part of a complex network of stress responses that, due to the impossibility of achieving a return to the original parameters of functioning, adjusts its functioning to adapt to the new conditions (a new homeorhetic state). We consider that it is mandatory to deepen the knowledge of the link between cognitive sequelae and a permanent low-grade activity of the inflammatory process. Mainly because this type of inflammation is related to the onset of several chronic-degenerative diseases and is evidence of chronic tissue stress conditions that limit the optimal development of the brain (47). Our next step is to focus on studying whether autonomic alterations of a vegetative type can also be related to chronic low-grade inflammation and whether changes in inflammatory molecules can be associated with the extent and location of perinatal damage assessed by CT imaging and neurophysiological studies.

Our study has some limitations, the first being related to the number of children studied. We classified our children in three groups depending on the magnitude of their perinatal risks and further divided them by the degree of neurological impairment, thus having very few cases for some analyses. Another limitation is the method used to determine the concentrations of the lipid mediators. The gold-standard is liquid chromatography coupled to mass spectrometry, and we used ELISA kits. Our children, however, have a detailed and well-documented follow-up and receive therapeutic interventions from a highly specialized multidisciplinary team.

## Conclusions

Consistent with our hypothesis, neurological signs that persist in preschool children with a history of perinatal risks were associated with modified patterns of serum molecules of the inflammatory process. Pro-inflammatory patterns were seen related to the presence and increased degree of clinical expression of the neurological dysfunction. These findings support the suggestion that persistent nervous system dysfunction may keep inflammatory responses active. This may happen even in the absence of an acute infection or damage process, and despite clinically successful neurodevelopmental interventions.

## Data Availability Statement

The raw data supporting the conclusions of this article will be made available by the authors, without undue reservation.

## Ethics Statement

The studies involving human participants were reviewed and approved by Comisión de Investigación y Comité de Etica. Instituto Nacional de Cardiología Ignacio Chávez. Written informed consent to participate in this study was provided by the participants’ legal guardian/next of kin.

## Author Contributions

MM, JE, RR-G, and RB contributed to conception and design of the study. MM and MB-P performed laboratory analyses and interpretation of results. MM, RR-G, and RB organized the database and performed the statistical analysis. MM and RB wrote the first draft of the manuscript. All authors contributed to the article and approved the submitted version.

## Funding

MM has received a scholarship from the National Council of Science and Technology (CONACyT), Mexico.

## Conflict of Interest

The authors declare that the research was conducted in the absence of any commercial or financial relationships that could be construed as a potential conflict of interest.
